# How Divorce and Parental Loss Shape Children’s Moral Growth and Emotional Resilience

**DOI:** 10.3390/bs15040539

**Published:** 2025-04-16

**Authors:** Fahri Sezer

**Affiliations:** Guidance and Psychological Counseling, Balikesir University, 10100 Balıkesir, Turkey; fahrisezer@balikesir.edu.tr

**Keywords:** divorce, adjustment, parental loss, children, moral maturity

## Abstract

(1) Background: This study investigates the relationship between the moral maturity levels of individuals who have experienced parental divorce or loss and their adjustment to the divorce process, considering various factors. (2) Methods: A total of 319 participants, aged between 10 and 18, who had either divorced parents or had experienced parental loss, were included in the study. Data were collected using the Adaptation to Divorce Inventory for Children and the Moral Maturity Scale. (3) Results: The results revealed that individuals who had experienced parental loss exhibited higher-than-average moral maturity levels. (4) Conclusions: A moderate, positive correlation was found between moral maturity and divorce adjustment, specifically in the areas of conflict resolution and depression–anxiety subscales. Additionally, substance use was associated with lower levels of moral maturity, while gender and educational level were significant predictors of moral maturity. These findings suggest that professional support, guidance programs, and family communication strategies are essential to promote the psychological adjustment of children affected by parental divorce or loss.

## 1. Introduction

The family plays a crucial role in the multifaceted development of children, encompassing their moral, psychological, and academic growth. Consequently, understanding the impact of divorce or parental loss on these domains is a significant area of research. Numerous studies have indicated that the experience of divorce is associated with increased levels of anxiety, depression, and behavioral difficulties in children (Brown, 2014; Cohen et al., 2014; Molgora et al., 2014; Sharp et al., 2022).

Divorce or the loss of a parent can significantly impact children’s mental health, often leading to emotional and psychological difficulties such as anxiety, depression, low self-esteem, and insecurity. These challenges may also hinder children’s ability to form healthy relationships and succeed academically in the long term (Levkovich & Eyal, 2021). Research has shown that children growing up without a father figure tend to experience lower self-esteem, while those living apart from either or both parents often struggle with anxiety, depression, phobias, shyness, and behavioral issues (Serin & Öztürk, 2007; Shariat et al., 2023). Furthermore, studies have highlighted that following parental separation, there is an increase in problems such as alcohol use, anxiety, and familial conflicts among children (Miralles et al., 2023).

Divorce or the loss of a parent can have a profound effect on children’s academic performance. Research has shown that divorce significantly impairs children’s educational outcomes and diminishes their ability to adapt socially (Potter, 2010). It has been linked to enduring negative consequences, including lower academic achievement, diminished self-confidence, and the emergence of behavioral issues (Amato & Keith, 1991; Kim, 2011). Additionally, children of divorced parents tend to exhibit lower levels of school motivation and develop less favorable relationships with their teachers (Sun & Li, 2011; Tyrone et al., 2024).

To better understand the impact of divorce on children, it is crucial to consider various socioeconomic and cultural factors. Numerous studies have highlighted that children from divorced families often experience academic challenges, primarily due to limited economic resources. Furthermore, factors such as reduced parent–child interaction and emotional instability at home can detrimentally affect children’s academic performance (McLanahan & Sandefur, 2009; Jeynes, 2012; Apata et al., 2023).

Cultural attitudes toward divorce also play a significant role, as in some societies, divorce is stigmatized and leads to social exclusion, while in others, it is viewed as more socially acceptable. These cultural differences influence children’s perceptions of the divorce process, their self-esteem, and their social relationships (Yip et al., 2015). Additionally, cultural norms and family structures shape the quality of post-divorce relationships between parents and children. In cultures where extended family ties are strong, children may receive emotional support from a broader network of relatives, whereas in more individualistic societies, such support may be more limited (Stack & Scourfield, 2015).

Changes in a family’s socioeconomic status can have a significant impact on children’s living conditions, educational opportunities, and the availability of psychosocial support following a divorce. Research indicates that children from economically disadvantaged families often experience higher levels of stress, academic challenges, and emotional difficulties (Sun & Li, 2011; Tyrone et al., 2024). These children may also face a diminished sense of belonging and increased uncertainty regarding their future as a result of parental separation (Furstenberg & Kiernan, 2001; Drill, 2021). Conversely, children from families with higher socioeconomic status are typically better equipped with resources and support mechanisms, which can help ease their adjustment during and after the divorce process (Chen et al., 2018; Neppl et al., 2016).

Traumatic events, such as divorce or the loss of a parent, can significantly influence the development of moral maturity in children (Ismail et al., 2022). These adverse experiences can affect the ways in which children understand and internalize core moral values, including empathy, responsibility, and justice. Factors such as reduced parental support, a diminished sense of trust, and family instability can complicate the development of these values. However, children who are able to navigate these challenges and grow up in a supportive, emotionally healthy environment may develop a more robust moral framework. In contrast, children who struggle to cope with the negative consequences of such events may experience lower levels of moral maturity during adolescence, which can hinder their ability to adapt to societal norms (Kogos & Snarey, 1995).

The environmental factors and psychological support play a critical role in the development of children following the loss of a parent or divorce. Research indicates that parental support and emotional security significantly facilitate children’s adjustment during this period, whereas a conflict-ridden divorce or the absence of emotional support may contribute to declines in moral values and the emergence of psychological difficulties (Amato & Keith, 1991; Bethell et al., 2017; Hashemi & Homayuni, 2017; Collins & Madsen, 2019). The aim of this study is to address the existing gap in the literature by exploring the multidimensional effects of divorce on children’s moral maturity and adaptation. Furthermore, it seeks to identify the support mechanisms necessary to assist children through this challenging process. The following research questions are proposed for investigation:

1. Does the adjustment to divorce among individuals with divorced parents differ based on their level of education, with potential gender differences between male and female individuals?
2. Is there a distinctive relationship between the adjustment to divorce and substance use among individuals with divorced parents?
3. Do male and female individuals with divorced parents exhibit differences in moral maturity based on their level of education?
4. How does substance use or non-use among individuals with divorced parents influence their moral maturity?
5. Which of the following factors—conflict-poor adjustment, depression–anxiety, and social support—serve as significant predictors of moral maturity in individuals with divorced parents?

## 2. Materials and Methods

### 2.1. Participants

The study was conducted with a sample of 319 participants, comprising 191 females (59.87%) and 128 males (40.13%), aged between 10 and 18 years. These individuals were enrolled in grades 5 through 12 and represented a demographic of children who had either experienced parental separation or the loss of one or both parents. The data were voluntarily provided by the participants with their informed consent. The required permissions for data collection were secured in collaboration with the school’s guidance and psychological counseling service. The psychological counselors informed the students and their parents about the confidentiality of the data through consent forms and ensured that the data collection process adhered to ethical guidelines. The research adhered to universal ethical standards and complied with the principles outlined in the 1975 Helsinki Declaration, as revised in 2000. The study was conducted with the approval of Balıkesir University Scientific Research Coordinatorship (protocol number 85048142-604.01.01/17/06-027).

### 2.2. Data Collection Tools

Moral Maturity Scale: In this study, the Moral Maturity Scale developed by Şengün and Kaya (2007) was utilized to assess the moral maturity levels of children who had experienced parental separation or loss. The scale is a five-point Likert-type instrument consisting of 66 items, designed to measure individuals’ moral maturity. The test–retest reliability coefficient of the scale was reported as 0.84, while the internal consistency (Cronbach’s alpha) was 0.93. The test–retest reliability coefficient for this study was 0.89, and the Cronbach’s alpha reliability coefficient calculated for this study was 0.922.

Divorce Adjustment Inventory: The Divorce Adjustment Inventory, developed by Portes et al. (1999) to assess individuals’ feelings and thoughts about divorce and the stress experienced due to it, was adapted into Turkish by Arifoğlu et al. (2010). The scale consists of three sub-dimensions: Conflict and Maladjustment, Depression and Anxiety, and Social Support. Items assessing the Conflict and Maladjustment and Depression and Anxiety dimensions include negatively worded statements. Lower scores in these sub-dimensions indicate better adjustment. In contrast, higher scores on the Social Support sub-dimension (which is reverse scored) reflect higher levels of adjustment to divorce. The scale measures the degree of a child’s adaptation to divorce, with higher scores indicating better adjustment. The Cronbach’s alpha values were calculated as 0.77 for the Conflict and Maladjustment subscale, 0.67 for the Depression and Anxiety subscale, and 0.63 for the Social Support subscale.

### 2.3. Analysis of Data

The underlying assumptions were thoroughly examined before conducting a Multivariate Analysis of Variance (MANOVA) to assess substance use and the joint effects of gender and education level on the adjustment of individuals whose parents had divorced, as well as a Univariate F analysis for the joint effects of gender and education level on moral maturity. Initially, correlations among the dependent variables were assessed to detect potential multicollinearity. The results indicated that the correlations between the variables ranged from r = −0.30 to r = 0.73 (see Table 5), suggesting acceptable levels of multicollinearity.

The Bartlett Test of Sphericity revealed a significant correlation between the dependent variables, with an approximate chi-square value of 4986.524 (*p* < 0.001). To assess linearity among the independent variables (gender, education level, and substance use status) and each pair of dependent variables, a scatterplot matrix was generated and carefully reviewed. Additionally, both univariate and multivariate normality were evaluated through Q-Q plots, and Mahalanobis distances were analyzed. The Kurtosis and Skewness values for each independent variable were found to be well within the acceptable range of ±1. Extreme values were further investigated through box plot analysis.

The analysis revealed the absence of extreme data points, with the Mahalanobis distance consistently exceeding 0.01. Furthermore, it was determined that there were no issues with multicollinearity, and linearity was observed between the variables. The data exhibited a normal distribution, and extreme values were not present. Given the sufficiently large sample size (*n* = 319), it was assumed that any potential violations of multivariate normality would have a minimal impact on Type I error. Additionally, the robustness of the F-test against deviations from normality supported the assumption of multivariate normality.

The Box’s M test results were reanalyzed to assess covariance equality, with Wilks’ Lambda applied when equality was assumed, and Pillai’s Trace used in the presence of inequality. Levene’s Test was conducted for each variable to evaluate the homogeneity of variances. In instances where differences between independent variables were observed, Tukey’s post hoc test was employed to identify the source of the differences. Moreover, when a significant interaction between independent variables (e.g., gender and education level) was detected, a simple effects analysis was performed (Huberty & Olejnik, 2006). Finally, discriminant analysis was carried out for each factor, and structure coefficients along with standardized function coefficients were examined to determine which dependent variable contributed to the group differences (Warner, 2012).

To assess the potential linear relationships between the variables, regression analysis was conducted, and binary combinations of the variables were examined through graphical representations (Kline, 2011). The results revealed a linear association between the variables. To evaluate homoscedasticity, the scatter plot of the dependent variable was inspected (Tabachnick & Fidell, 2007). It was found that the error term (residuals) exhibited consistent variance across all levels of the independent variables, with no cone-shaped distribution indicating heteroscedasticity. Additionally, an analysis confirmed the absence of multicollinearity (Kline, 2011), as the correlations were moderate in strength between the dependent and independent variables, as well as among the independent variables themselves.

To examine the predictors of moral maturity, a path analysis based on observed variables was conducted, focusing on dimensions of Adjustment to Divorce. The analysis first tested the structural model. The measurement model included 88 reflective indicators, corresponding to four latent factors that represented the key constructs outlined in the study. To assess the measurement model, both convergent and discriminant validity were evaluated, with the Fornell and Larcker (1981) criteria applied. Additionally, Cronbach’s alpha values were calculated to assess the reliability of the scale.

The results indicated that the model fit indices for both Adjustment to Divorce (chi-square/df = 2.13, RMSEA = 0.045, IFI = 0.99, NFI = 0.99, NNFI = 0.99, SRMR = 0.042, CFI = 0.99) and Moral Maturity (chi-square/df = 2.73, RMSEA = 0.055, IFI = 0.98, NFI = 0.98, NNFI = 0.97, SRMR = 0.056, CFI = 0.96) demonstrated adequate to excellent fit, as per the criteria outlined by Hu and Bentler (1999), Kline (2011), and Tabachnick and Fidell (2007). Furthermore, the Composite Reliability (CR) values, assessed for both Convergent and Discriminant Validity, ranged from 0.89 to 0.93, with all values exceeding the acceptable threshold of 0.75. The Average Variance Extracted (AVE) values for all variables ranged from 0.42 to 0.55. While an AVE value of 0.50 or higher is typically recommended, Fornell and Larcker (1981) and Kandemir et al. (2019) suggest that if the BG coefficient is above 0.70, an AVE value of 0.40 or above is considered acceptable.

The square roots of the average variances exceed the correlations between the scale factors, indicating that the structural model effectively meets the criteria for both convergence and discriminant validity. Reliability analysis, as assessed by Cronbach’s Alpha, yielded the following values: 0.92 for the Moral Maturity dimension, 0.84 for the Conflict-Poor Adjustment dimension of the Divorce Adjustment scale, 0.78 for Depression and Anxiety, and 0.67 for the Social Support dimension. Data analysis was conducted using IBM SPSS 24.0 and Jamovi 2.3.8 software.

## 3. Results

### 3.1. Descriptive Analysis

The descriptive analysis findings related to the moral maturity and adaptation to divorce of the participants in the study are presented in Table 1.

The analysis reveals that individuals with divorced parents exhibited a relatively high level of Moral Maturity (M = 4.21, SD = 0.45). In contrast, the overall mean score on the Adjustment to Divorce scale for these individuals was low (M = 3.25, SD = 0.47). Specifically, within the sub-dimensions of the Adjustment to Divorce scale, the Conflict and Maladjustment sub-dimension had a notably high average score (M = 3.91, SD = 3.86). Conversely, both the Depression and Anxiety (M = 3.13, SD = 0.89) and Social Support (M = 2.72, SD = 0.65) sub-dimensions showed lower mean scores.

### 3.2. Analysis with Respect to Mutual Effect of Both Gender and Education Level According to Adjustment to Divorce

A Multivariate Analysis of Variance (MANOVA) was conducted to assess whether individuals with divorced parents exhibited differences based on Gender and Education Level (Gender x Education Level interaction). The Box’s M test yielded a value of 21.703 (F = 1.181, *p* > 0.05), indicating that the covariance matrices across the groups were homogeneous. Additionally, Levene’s Test was employed to examine the homogeneity of variances for the dependent variables.

The assumption of homogeneity of variances was confirmed for each of the subscales: Conflict and Maladjustment (F = 2.380, *p* > 0.05), Depression and Anxiety (F = 0.475, *p* > 0.05), Social Support (F = 1.362, *p* > 0.05), Parenting (F = 2.468, *p* > 0.05), Communication (F = 2.311, *p* > 0.05), and Social Support (F = 1.065, *p* > 0.05). Given the equality of covariance matrices and homogeneity of variances, Wilks’ Lambda (λ) was employed as the multivariate test. Results indicated that the dependent variables were significantly influenced by Gender (Wilks’ λ = 0.807, F(3, 313) = 25.000, *p* < 0.001, η^2^ = 0.193) and Level of Education (Wilks’ λ = 0.264, F(3, 313) = 290.915, *p* < 0.001, η^2^ = 0.736), as well as by the Gender × Level of Education interaction (Wilks’ λ = 0.867, F(3, 313) = 16.052, *p* < 0.001, η^2^ = 0.133). Given the large Partial Eta Squared values, the MANOVA analysis proceeded as planned.

The results of the MANOVA analysis reveal significant differences across all three dimensions with respect to gender. Specifically, women exhibited lower mean scores than men in the Conflict and Maladjustment and Depression and Anxiety subscales. Conversely, men demonstrated lower mean scores in the Social Support sub-dimension. Regarding education level, significant differences were also observed across all three dimensions. In the Conflict and Maladjustment and Depression and Anxiety subscales, individuals studying in secondary schools had lower mean scores compared to those in middle schools. However, in the Social Support sub-dimension, students in secondary schools reported higher mean scores than their counterparts in middle schools. Furthermore, a significant interaction effect between Gender and Education Level was identified across all dimensions. A one-way discriminant analysis was conducted for males, and then a separate one way discriminant analysis was conducted for females, with education level as a single factor. Descriptive Discriminant Analysis (DDA) was applied to determine group differences. DDA results for both males and females, according to education level, are shown Table 2.

Discriminant function analysis (DFA) was conducted to examine group differences based on gender. The results of the DFA are presented in Table 2 for both women and men. For women, Function 1 (λ = 0.243, *p* < 0.05) was statistically significant, and for men, Function 1 (λ = 0.261, *p* < 0.05) also demonstrated significance. When the canonical correlation was translated into a percentage, it was found to be 76% for women and 74% for men. Further analysis of the standardized coefficients revealed that for women, the most influential variable was “Conflict and Maladjustment”, followed by “Depression and Anxiety”. In contrast, for men, “Depression and Anxiety” emerged as the primary factor, with “Conflict and Maladjustment” following. These findings suggest that these variables contributed most significantly to the observed gender differences in educational level. Analysis of group centroids showed that female students had lower mean scores compared to male students at both the middle and high school levels.

### 3.3. Adjustment to Divorce in Terms of Substance Use

A MANOVA was conducted to examine whether individuals with divorced parents differed in terms of substance use based on their adjustment to the divorce. Prior to analysis, covariance equality was assessed using Box’s M test, which yielded a value of 2.715 (*p* > 0.05), indicating that the covariance matrices were equal across groups. The Levene Test was then applied to assess the homogeneity of variances for the dependent variables. The results demonstrate that the variances were homogeneous for each of the subscales: Conflict and Maladjustment (F = 0.059, *p* > 0.05), Depression and Anxiety (F = 1.667, *p* > 0.05), and Social Support (F = 1.079, *p* > 0.05).

Since the covariance matrices between the groups were not homogeneous, Wilks’ Lambda was applied to assess the data. The results of the Wilks’ Lambda test indicate that the dependent variables were significantly influenced by substance use (Wilks λ = 0.928, F(3, 315) = 8.139, *p* < 0.001, η^2^ = 0.072). Given the sufficiently large Partial Eta Squared values, a Multivariate Analysis of Variance (MANOVA) was conducted. The MANOVA revealed significant differences in the dimensions of “Conflict and Maladjustment” (F(1, 317) = 20.250, *p* < 0.05) and “Depression and Anxiety” (F(1, 317) = 12.522, *p* < 0.05) related to substance use, while no significant difference was found in the dimension of “Social Support” (F(1, 317) = 0.169, *p* > 0.05). To further explore group differences, a Discriminant Function Analysis (DFA) was performed, with the results presented in Table 3 according to education level.

The results indicate that non-substance users exhibit higher mean scores in both the Conflict and Maladjustment dimensions, as well as in the Depression and Anxiety sub-dimensions. Descriptive Discriminant Analysis (DDA) revealed that only one function (λ = 0.939) was significant (*p* value < 0.05). To further evaluate individuals’ divorce adjustment scale scores, standardized discriminant function coefficients and structure coefficients were examined (Table 3). The relative absolute for SCDF was used to determine the relative importance of independent variables in predicting substance use or not. According to Table 3, the best independent variables in predicting the dependent variable are noted in Conflict and Maladjustment (0.897), thus indicating that ratings on these factors made the largest contributions in discriminating between substance use or not. Furthermore, the square of the canonical correlation (0.2462), converted to a percent, indicates about 6% of variation for substance use.

### 3.4. Analysis with Respect to Mutual Effect of Both Gender and Education Level According to Moral Maturity

A univariate F-test was conducted to examine the joint effects of gender and education level on individuals’ moral maturity. The results revealed that both gender (F = 152.597, *p* < 0.001, H^2^ = 0.326) and education level (F = 144.460, *p* < 0.001, H^2^ = 0.314) had significant effects on moral maturity. Specifically, men exhibited higher levels of moral maturity than women. Additionally, individuals enrolled in high school demonstrated higher moral maturity compared to those attending middle school. The univariate F-test results are shown Table 4.

The analysis reveals that the interaction between gender and education level significantly influences moral maturity (F = 90.661, *p* < 0.001). Specifically, women at the secondary school level exhibit a lower average moral maturity score compared to their male counterparts at the same level, as well as both male and female students at the middle school level. These findings suggest a notable disparity in moral maturity based on the combined effects of gender and educational attainment.

### 3.5. Analysis of Moral Maturity in Terms of Substance Use

An independent samples *t*-test was conducted to examine whether moral maturity levels differed between individuals with separated parents who used substances and those who did not. The analysis revealed that the mean moral maturity score for substance users (M = 3.39, SD = 0.37) was significantly lower than that of non-substance users (M = 4.25, SD = 0.45). The *t*-test statistic was found to be *t*(158) = −3.738, *p* < 0.001, with a Cohen’s d of 0.63, indicating a medium to large effect size. These results suggest that substance use is significantly associated with lower levels of moral maturity among individuals with separated parents.

### 3.6. Correlational Analysis Between Moral Maturity and Divorce Adjustment

A correlation analysis was performed to examine the potential relationship between individuals’ level of adaptation to divorce and their moral maturity. The findings of the analysis are summarized in Table 5.

The analysis revealed a positive and moderately significant relationship between Moral Maturity and the Conflict and Maladjustment (r = 0.66, *p* < 0.05) and Depression–Anxiety (r = 0.49, *p* < 0.05) sub-dimensions of the Adjustment to Divorce scale. Additionally, a negative and moderately significant relationship was found between Moral Maturity and the Adjustment to Divorce Social Support sub-dimension (r = −0.30, *p* < 0.05).

### 3.7. Path Analysis Results

**Figure 1:**
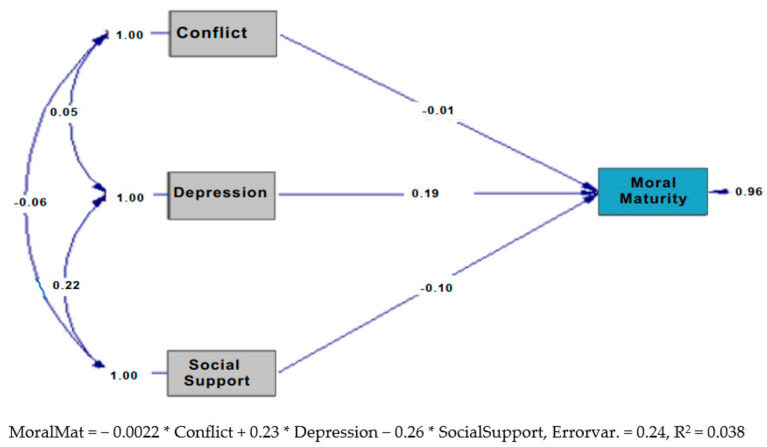
Path diagram.

An analysis was conducted to explore the relationship between the adjustment levels of individuals with divorced parents and their moral maturity. As a result of the analysis, the model demonstrated an excellent fit. The fit indices were as follows: RMSEA = 0.000, chi-square *p*-value = 1.000, with all other indices also indicating an excellent model fit. The standardized path diagram of the model is presented in Figure 1.

The analysis reveals that the Conflict and Maladjustment variable did not significantly contribute to the model (*t* = −0.17, *p* > 0.05). In contrast, Depression and Anxiety (*t* = 3.33, *p* < 0.05) and Social Support (*t* = 2.01, *p* < 0.05) were found to make significant contributions. Specifically, depression and anxiety were positively associated with moral maturity, while social support was negatively associated with it. The regression model can be expressed as follows: MoralMaturity = −0.0022 * Conflict + 0.23 * Depression − 0.26 * SocialSupport, Errorvar. = 0.24, R^2^ = 0.038.

## 4. Discussion

The findings of the study reveal that individuals who experienced parental separation or loss exhibited moral maturity levels above the general average. However, the overall low average score on the Adaptation to Divorce Scale indicates that these individuals face challenges in adjusting to the divorce process. Notably, higher scores were recorded in the Conflict and Maladjustment and Depression and Anxiety sub-dimensions of the scale, compared to the Social Support sub-dimension. The lowest score observed in the Social Support sub-dimension suggests a lack of sufficient social support mechanisms, which may hinder children’s ability to adapt effectively to the divorce. While previous research has shown that high social support scores are associated with better adaptation to divorce (Şengün & Kaya, 2007), our study found that participants reported low levels of social support. These findings suggest that individuals experience difficulty in adjusting to divorce and endure negative emotional consequences. Furthermore, the lack of adequate support from their social environment exacerbates the challenges they face in coping with the psychological effects of divorce.

The findings of this study align with existing literature indicating that individuals who experience parental loss or divorce may exhibit increased levels of moral maturity. Research suggests that facing crises can foster the development of moral traits such as a heightened sense of responsibility and empathy (Amato, 2000; Tullius et al., 2022). Similarly, previous studies have demonstrated that early exposure to family difficulties can enhance moral development by promoting self-awareness (Masten, 2001; Toth & Cicchetti, 2013; Elder, 2018). Hetherington (2003) further argues that individuals who experience parental loss or divorce may assume greater responsibilities at an early age and adopt a more cautious approach to social relationships.

Research on individuals with low levels of adjustment and those exposed to high levels of family conflict indicates that these individuals are more likely to experience emotional difficulties. Studies have shown that the risk of depression and anxiety significantly increases when intense family conflict is compounded by a lack of social support (Taylor, 2011; Sharp et al., 2022). These findings highlight the negative impact of divorce and parental loss on psychological adjustment, particularly for individuals with limited access to adequate support systems, resulting in lower emotional resilience. However, it is important to acknowledge that parental loss or divorce can have both positive and negative effects on individuals. While exposure to crisis situations may foster the development of moral traits such as responsibility and empathy, the presence of psychological and social support mechanisms remains crucial in facilitating the adaptation process.

The research findings highlight the significance of interactions between gender, education level, and psychosocial variables. Notably, the lower mean scores of female students on the Conflict and Poor Adjustment and Depression and Anxiety subscales, compared to their male counterparts, suggest gender-based differences in psychological resilience and coping strategies. Existing literature indicates that women generally tend to seek higher levels of social support, which may contribute to their greater psychological resilience compared to men (Taylor, 2011; Cohen et al., 2014; Torres & Garcia, 2019). It has been emphasized that women are more likely to use social support as a coping mechanism, which in turn may enhance their psychological resilience (Scholte et al., 2001; Tamres et al., 2002).

In parallel, the lower mean scores observed for male students in the “Social Support” sub-dimension may reflect challenges in accessing social networks or utilizing social support mechanisms (Scholte et al., 2001). However, some studies have found that women tend to exhibit lower levels of resilience than men following potentially traumatic events (Bonanno et al., 2007). On the other hand, other research suggests that there are no significant gender differences in resilience levels, with factors such as personality traits and social support potentially influencing this outcome (Campbell-Sills et al., 2009). These differences can be attributed to socialization processes and traditional gender roles. Women are generally socialized to express their emotions and seek social support, which may enhance their psychological resilience through the development of strong social networks. In contrast, men are often conditioned to suppress their emotions and rely on self-reliant coping strategies. While this may bolster resilience in certain contexts, it may also limit the development of adaptive coping skills (Amoadu et al., 2024).

The observed differences in psychological adjustment between middle school and high school students highlight the influence of developmental and age-related factors. Middle school students report lower levels of “Conflict and Maladjustment” and “Depression and Anxiety” compared to their high school counterparts, which may reflect the heightened academic and social pressures associated with the high school experience (Steinberg, 2005). Furthermore, the higher levels of perceived social support among middle school students can be attributed to the relatively greater effectiveness of family and teacher support during this developmental stage (Eccles & Roeser, 2011). Conversely, the reduced family support observed during high school may exacerbate adolescent challenges, contributing to increased psychological distress. The pronounced role of the “Conflict and Maladjustment” and “Depression and Anxiety” variables in the interaction between gender and education level underscores the significance of individual differences in psychosocial well-being. These findings reinforce the need for targeted interventions tailored to the specific developmental and gender-based needs of adolescents to promote psychological adjustment and mental health (Hankin et al., 2007).

Research findings indicate that individuals who abstain from substance use exhibit higher mean scores on the conflict–maladjustment and depression–anxiety subscales. This suggests that substance use may adversely affect individuals’ capacity to adapt to the divorce process and regulate their emotions. Previous literature has established that substance use undermines stress-coping mechanisms (Sinha, 2008; Wills, 2013; Lyness & Fischer, 2021) and is closely linked to maladaptive behaviors (Glantz & Pickens, 1992; Low et al., 2012). The analysis revealed that conflict and maladjustment factors are the most significant predictors of substance use. This finding underscores the potential role of interpersonal conflict and maladaptive experiences as critical risk factors associated with substance use. Furthermore, research has shown that individuals from disrupted family environments may resort to substance use as a means of addressing emotional voids and alleviating psychological distress (J. F. Kelly et al., 2016; Lyness & Fischer, 2021). These individuals may exhibit greater vulnerability to substance use (Umberson & Karas Montez, 2010; Cohen et al., 2014). Importantly, substance use should not be viewed solely as an individual issue but rather as one influenced by a dynamic interplay of environmental and psychosocial factors (Sinha, 2008; Yip et al., 2015; J. F. Kelly et al., 2016; Sharp et al., 2022).

The analysis reveals that gender and educational attainment significantly influence individuals’ levels of moral maturity. The finding that men exhibit higher moral maturity averages than women aligns with prior literature suggesting that gender exerts differential effects on moral development (Gibbs et al., 2007; Torres & Garcia, 2019; Apata et al., 2023). According to Gilligan’s (1993) theory of moral development, women tend to adopt a relational and responsibility-centered approach to moral decision making, whereas men are more inclined to operate within a framework emphasizing justice and rules. This distinction offers valuable insights into the underlying reasons for the observed gender differences in moral maturity.

Research findings indicate a significant relationship between education level and moral maturity, aligning with prior studies that suggest moral maturity tends to increase with higher levels of education (Rest et al., 1999; Walker, 2006; Ismail et al., 2022). Specifically, individuals with a high school education demonstrate higher levels of moral maturity compared to those with only a middle school education, supporting the literature that posits moral reasoning skills become more complex with age (Kohlberg, 1984; Kohlberg & Gilligan, 2014). However, the observation that women with a middle school education exhibit lower average moral maturity than other groups highlights the potential interaction of age and gender. This finding is consistent with research suggesting that early adolescent females may experience developmental challenges that temporarily impede moral maturity (Arnett, 1999; Smetana, 2010; Miralles et al., 2023). These results underscore that the development of moral maturity is influenced by the interplay of gender, age, and educational attainment, each contributing unique dynamics to this complex process.

Research findings indicate that individuals who engage in substance use exhibit significantly lower levels of moral maturity compared to non-users. This evidence suggests that substance use adversely impacts moral development, diminishing the capacity to adhere to social values and norms. The existing literature highlights that substance use impairs ethical decision-making processes, self-control abilities, and empathy levels (Rest et al., 1999; Shariat et al., 2023). Notably, the “Four Component Model” of moral development underscores the cognitive, emotional, and behavioral dimensions of moral maturity and posits that these processes are susceptible to disruption by risky behaviors such as substance use (Rest et al., 1999). Furthermore, the detrimental effects of substance use on emotional regulation and moral reasoning are linked to neurological alterations in the prefrontal cortex and limbic system (Volkow et al., 2011). These changes contribute to a decline in fundamental moral capacities, including empathy, responsibility, and ethical reasoning, among individuals who use substances (Kohlberg, 1984). However, a low level of moral maturity may hinder an individual’s adaptation to social norms and increase the likelihood of engaging in risky behaviors such as substance use (Wallace et al., 2007).

The findings of this study demonstrate a positive and moderately significant relationship between moral maturity and the sub-dimensions of conflict, poor adjustment, and depression–anxiety in the context of divorce. This suggests that individuals with higher levels of moral maturity are more likely to experience heightened emotional challenges following divorce, including increased conflict, maladjustment, depression, and anxiety. Moral maturity, which is closely tied to individuals’ values, beliefs, and ethical standards, appears to influence the psychological difficulties encountered during the post-divorce period (Stanley, 1978; Tangney et al., 2007; Kołodziej-Zaleska & Przybyła-Basista, 2016; Sharp et al., 2022). Furthermore, the study identifies a negative association between adjustment to divorce and social support, underscoring the critical role of social support in mitigating emotional distress during the divorce process. Conversely, insufficient social support may exacerbate psychological challenges and hinder emotional recovery (Amato, 2000; J. B. Kelly & Emery, 2003).

## 5. Conclusions

Providing professional psychological support to assist with the psychological and emotional adjustment of children and families during and after the divorce process is of paramount importance. Amato (2000) highlighted the critical role of parents in maintaining open communication with their children and offering emotional support to safeguard their emotional well-being post-divorce. In this regard, it would be advantageous for educational institutions to establish social support groups and counseling programs tailored to children from divorced families. Stolberg and Mahler (1994) demonstrated that school-based interventions are effective in alleviating the stress experienced by children following a divorce. Furthermore, there is a need to develop support mechanisms that address the unique psychological needs of children on an individual basis.

It is crucial for divorced parents to receive training in communication and parenting skills to support their children’s adjustment to the divorce process. An example of an evidence-based intervention program is the “New Beginnings Program” (Wolchik et al., 2000), which assists divorced parents in enhancing communication with their children and improving their parenting abilities. This program seeks to mitigate children’s internalizing and externalizing behaviors by equipping parents with essential tools, such as active listening techniques, strategies to promote positive family interactions, and effective discipline methods.

Furthermore, it is essential to develop preventive programs aimed at mitigating the risk of substance use among adolescents, while also establishing financial assistance and social support mechanisms to alleviate the economic challenges faced by families. Amato and Gilbreth (1999) emphasized that economic stability plays a critical role in the well-being of children following a divorce. State-supported social assistance programs and counseling services can significantly aid families in navigating the transition process, providing them with the necessary support to adapt to new circumstances. Such support will facilitate a healthier adjustment for both children and parents in the post-divorce period.

The psychological impact of divorce on children is significantly influenced by the cultural context in which it occurs. In Western societies, divorce is often viewed as a more normalized social phenomenon, supported by values of individuality and autonomy. Consequently, children’s emotional reactions tend to be short term, typically manifesting as temporary feelings of anxiety, anger, or sadness (Amato, 2000). In contrast, Eastern societies, where collectivist cultural values and strong family ties prevail, tend to view divorce as a more stigmatizing experience. This perception can lead to more enduring psychological effects in children, such as feelings of guilt, social isolation, and identity confusion (Amato & Keith, 1991; Fabricius & Luecken, 2007; Park & Lau, 2016). These cultural differences underscore the significant role that cultural norms and lifestyles play in shaping both the perception of divorce and the process of children’s adaptation.

This study provides a significant contribution to understanding the unique needs and dynamics of specific groups. While the inclusion of only children from disrupted families allows for an in-depth examination of the dynamics unique to this population, it also imposes certain limitations on the generalizability of the findings. The sample size was constrained by the specific criteria of including children from divorced families who were enrolled in middle and high school. Therefore, future research incorporating larger and more diverse samples, as well as cross-cultural comparisons, would offer a more comprehensive and nuanced perspective on the adaptation processes and moral development of children from divorced families.

## Figures and Tables

**Table 1:** Descriptive statistics (N = 319).

		N	AD: Conflict and Maladjustment M (SD)	AD: Depression and Anxiety M (SD)	AD: Social Support M (SD)	Moral Maturity M (SD)
Gender	(a) Female	191	3.71 (0.89)	2.93 (0.75)	2.86 (0.64)	4.06 (0.51)
	(b) Male	128	4.21 (0.70)	3.42 (0.98)	2.50 (0.62)	4.43 (0.31)
Level of Education	(c) Middle School	110	2.97 (0.57)	2.18 (0.49)	3.05 (0.60)	3.85 (0.46)
	(d) High School	209	4.41 (0.48)	3.63 (0.61)	2.54 (0.62)	4.40 (0.33)
Substance Use	(e) Yes	48	3.41 (0.86)	2.71(94)	2.75 (0.63)	3.99 (0.37)
	(f) No	271	4.00 (0.83)	3.20 (0.86)	2.71 (0.66)	4.25 (0.43)
Overal		319	3.91 (0.86)	3.13 (0.89)	2.72 (0.65)	4.21 (0.45)
	Skewness	319	−0.591	−0.166	0.119	−0.544
	Kurtosis	319	−0.737	−0.812	−0.397	−0.222

AD: Adjustment to Divorce, M = Mean, SD = Stand. Deviation.

**Table 2:** Discriminant analysis for females and males according to education level.

	Function at Group Centroids			CC	SCDF (SM)		
	f	Middle School	High School		Conflict and Maladjustment	Depression and Anxiety	Social Support
Female	1	−2.284	−2.524	0.870	0.795 (0.877)	0.502 (0.649)	0.123 (−0.182)
Male	1	1.351	1.106	0.860	0.454 (0.899)	0.719 (0.362)	−0.051 (−0.303)

f = Function; CC = Canonical Correlation; SCDF = Standardized Canonical Discriminant Function Coefficients; SM = Structure Matrix.

**Table 3:** Discriminant analysis results.

DVs	MS	F	Sig.	η^2^	Differences	SCDF1 (SM1)	CC
Conflict and Maladjustment	14.176	20.250	0.000 *	0.06	Substance Non-Users > Users	0.897 (0.90896)	0.246
Depression and Anxiety	9.698	12.522	0.000 *	0.04	Substance Non-Users > Users	0.136 (0.71483)	
Social Support	0.074	0.169	0.682	-	-		

MS = Mean Square; CC = Canonical Correlation; SCDF = Standardized Canonical Discriminant Function Coefficients; SM = Structure Matrix. * *p* < 0.05.

**Table 4:** Univariate F-test results.

	MS	df	F	*p*	Tukey	H^2^
Gender	13.657	1	152.597	0.000	b > a	0.326
Education Level	12.928	1	144.460	0.000	d > c	0.314
Gender x Level of study	8.114	1	90.661	0.000	bd, bc, ad > ac	0.223
Error	0.089	315				

bd = Male High School, bc = Male Middle School, ad = Female High School, ac = Female Middle School.

**Table 5:** Correlational analysis.

	AD: Conflict and Maladjustment	AD: Depression and Anxiety	AD: Social Support
AD: Conflict and Maladjustment	1		
AD: Depression and Anxiety	0.733 *	1	
AD: Social Support	−0.456 *	−0.462 *	1
Moral Maturity	0.665 *	0.492 *	−0.297 *

* *p* < 0.01.

## Data Availability

The data that support the findings of this study are available from the corresponding author upon reasonable request.
